# Development and Evaluation of Bilayer Sustained-Release Tablets of Ruxolitinib Using Discriminative Pharmacokinetic Analysis and IVIVC

**DOI:** 10.3390/pharmaceutics17040432

**Published:** 2025-03-28

**Authors:** Namhyuck Kim, Kyoungho Kim, Seungwei Jeong, Jiyeong Kim, Helen Cho, Young-Joo Lee, Sangyeob Park

**Affiliations:** 1 Samyang Holdings Corp., 295 Pangyo-ro, Bundang-gu, Seongnam-si 13488, Republic of Korea; 2 Division of Biopharmaceutics, College of Pharmacy, Kyung Hee University, 1 Hoegi-dong, Dongdaemun-gu, Seoul 02453, Republic of Korea; 3 Department of Integrated Drug Development and Natural Products, Kyung Hee University, 1 Hoegi-dong, Dongdaemun-gu, Seoul 02453, Republic of Korea

**Keywords:** bilayer tablet, sustained release formulation, ruxolitinib, IVIVC, dissolution

## Abstract

**Objectives**: This study explores the development and evaluation of a bilayer sustained-release (SR) tablet formulation of ruxolitinib. As a BCS Class 1 drug, ruxolitinib requires twice-daily dosing due to its short half-life. We designed a bilayer tablet that integrates immediate-release (IR) and SR components in varying ratios to achieve sustained plasma concentrations, which we evaluated using discriminative analysis. **Methods**: Bilayer tablets combining IR and SR components were prepared in different ratios. In vitro dissolution tests and pharmacokinetic studies were conducted using Beagle dogs, followed by the evaluation of in vivo–in vitro correlation (IVIVC), along with a discriminative pharmacokinetic analysis focused on the SR layer. **Results**: A discriminative pharmacokinetic and IVIVC analysis was applied to all bilayer tablets, offering clearer insights into the plasma concentration and dissolution profiles. Pharmacokinetic studies showed that test formulation F4, which has a 20:20 IR-to-SR ratio, is expected to provide a similar area under the curve (AUC) while prolonging exposure compared to the reference IR tablet. **Conclusions**: This study highlights the potential of a bilayer tablet approach, combined with discriminative pharmacokinetic and IVIVC analysis, for creating a sustained-release dosage form of ruxolitinib.

## 1. Introduction

Ruxolitinib, marketed under the brand name Jakavi^®^ (Novartis Pharmaceuticals Corporation, Basel, Switzerland), is a potent and selective Janus Kinase (JAK) 1 and 2 inhibitor approved for the treatment of myelofibrosis and polycythemia vera [1]. As a potent and selective inhibitor of Janus Associated Kinases (JAK1 and JAK2), Jakavi^®^ has demonstrated significant efficacy in alleviating disease-related symptoms and improving the quality of life for patients [2].

Jakavi^®^ is classified as a Biopharmaceutics Classification System (BCS) Class 1 drug, characterized by high solubility and high permeability. Ruxolitinib is rapidly absorbed after oral administration, with a time to maximum concentration (Tmax) of approximately 1 h [3,4]. The drug is extensively metabolized, primarily by CYP3A4, and mainly excreted as metabolites, with less than 1% of the dose recovered as an unchanged drug in urine [4]. It exhibits dose-proportional pharmacokinetics and has a relatively short half-life of 3 h [4], necessitating the development of sustained release formulation [5].

Despite these favorable pharmacokinetic properties, the current immediate-release (IR) formulation of Jakavi^®^ requires twice-daily dosing due to its short half-life [6]. This dosing regimen can lead to challenges in patient adherence and potentially suboptimal therapeutic outcomes, particularly in chronic conditions that require long-term treatment [7]. For ruxolitinib, a well-designed sustained-release (SR) formulation could potentially reduce dosing frequency, minimize peak-to-trough fluctuations in plasma concentrations, and thereby enhance patient convenience and compliance while maintaining or even improving efficacy [3,8].

For BCS Class 1 drugs like ruxolitinib, the application of pharmacokinetic and in vitro-in vivo correlation (IVIVC) analysis can be valuable in optimizing SR formulations, as it allows for the exploration of various release profiles and their potential impact on systemic exposure without the need for extensive in vivo studies [9]. IVIVC enabled us to establish a relationship between in vitro drug dissolution or release and in vivo drug absorption or exposure [10]. For BCS Class 1 drugs, the development and application of IVIVCs present both opportunities and challenges. On the one hand, the high solubility and permeability of these drugs can simplify the establishment of correlations between dissolution and absorption. On the other hand, the rapid and complete absorption typical of BCS Class 1 drugs may limit the discriminatory power of traditional IVIVC approaches, necessitating careful consideration of formulation strategies and dissolution testing conditions [11]. A bilayer tablet approach offers several advantages compared to other SR formulations. Bi-layer tablets allow for a combination of IR layer to provide a rapid onset of action and SR layer to maintain therapeutic plasma concentrations over a prolonged period, enhancing therapeutic effects and patient compliance [12,13,14,15]. Such a formulation strategy could address the pharmacokinetic limitations of the current IR formulation while capitalizing on the favorable BCS Class 1 properties of ruxolitinib.

The present study aims to develop an IR and SR bilayer formulation of ruxolitinib as a sustained dosage form of the current ruxolitinib IR formulation, Jakavi^®^. By utilizing a discriminative pharmacokinetic and IVIVC analysis using Beagle dog, we seek to optimize the IR and SR ratio of a bilayer tablet formulation. This approach allows for the rational design of a formulation that meets the desired pharmacokinetic profile while minimizing the need for extensive in vivo testing during the development phase.

## 2. Materials and Methods

### 2.1. Materials

Ruxolitinib phosphate was purchased from MSN Laboratories Pvt. Ltd. (Telangana, India). Microcrystalline cellulose (HW101^®^, JRS pharma, Rosenberg, Germany), lactose monohydrate (Supertab^®^ 11SD, DFE pharma, Goch, Germany), sodium starch glycolate (Roquette, Lestrem, France), povidone (Kollidon^®^ 30, BASF, Ludwigshafen, Germany), hydroxypropyl cellulose (HPC-L^®^, Nippon soda, Tokyo, Japan), polyethylene glycol 6000 (Vasudha chemicals, Mumbai, India), magnesium stearate (FACI), and colloidal silicon dioxide (SYLOID^®^ 244FP) were purchased from IMCD Korea (Seoul, Republic of Korea). Polyethylene oxide (Polyox™, Dupont/IFF, Wilmington, NC, USA), which has various molecular weights (POLYOX WSR 205, POLYOX WSR N60K), and Opadry^®^ coating agent (Opadry^®^ 20A, Opadry^®^ 200F) were purchased from Colorcon Korea (Suwon, Republic of Korea). The reference standard of ruxolitinib phosphate was received from MSN Laboratories Pvt. Ltd. (Telangana, India), and ruxolitinib-d9 was purchased from Clearsynth Labs Ltd. (Maharashtra, India) Unless otherwise indicated, all other chemicals were of analytic or high-performance liquid chromatography (HPLC) grade.

### 2.2. Preparation of Bilayer SR Tablet

The formulations were designed as bilayer tablets combining IR and SR components in varying ratios: 5:35 (test formulation F1), 10:30 (test formulation F2), 15:25 (test formulation F3), and 20:20 (test formulation F4). Each layer has its separate granulation process. For the preparation of IR granules, ruxolitinib phosphate, microcrystalline cellulose, lactose monohydrate, and sodium starch glycolate were mixed with a binding solution, which is povidone and hydroxypropyl cellulose dissolved in purified water, using Simens a TP G Lab high shear mixer (COMASA, Buenos Aires, Argentina). After mixing, the granules were dried using an FO-600M Drying Oven (JEIO TECH, Daejeon, Republic of Korea). Dried granules were sized using the 1.0 mm mesh and lubricated with magnesium stearate and colloidal silicon dioxide. For the preparation of SR granules, ruxolitinib phosphate, polyethylene oxide, polyethylene glycol 6000, and hydroxypropyl cellulose were passed through the 1.0 mm mesh and mixed using V-mixer (DOTT Bonapace and C V-MIX MP6C, Cusano Milanino, Italy). After mixing, the granules were lubricated with magnesium stearate.

**Figure 1:**
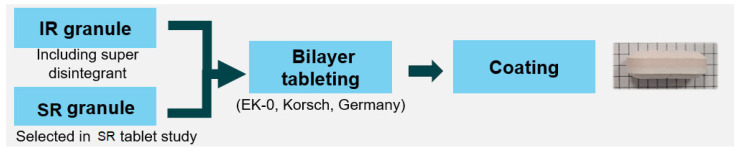
Manufacturing scheme of ruxolitinib bilayer tablet.

The granules were compressed to form bilayer tablets consisting of IR and SR layers using an AutoTab-200TR tablet press (ICHIHASHI SEIKI, Kyoto, Japan) using oval shape punches. The compaction pressure was 10 kN, and the average hardness of tablets was 20–25 kp, respectively. The batch size was 200 tablets per formulation. After forming bilayer tablets, the tablets were coated with Opadry^®^ coating agent using a ighcoater^®^ (Freund Bldg, Tokyo, Japan). The detailed compositions of each formulation are shown in Table 1. The manufacturing scheme is shown in Figure 1.

### 2.3. In Vitro Dissolution Tests

The in vitro drug release studies were carried out using the USP apparatus II (PTWS1210 dissolution apparatus and DSR-M13 sampling station, Pharma Test Apparatebau AG, Hainburg, Germany) at a paddle rotation speed of 50 rpm. The release patterns of the reference (Jakavi^®^, Novartis Pharma stein AG, Stein, Switzerland) were confirmed in various media (0.1 M HCl (pH 1.2), 0.05 M acetate buffer (pH 4.0), 0.2 M phosphate buffer (pH 6.8), and distilled water, as stipulated in the Korean Pharmacopoeia) maintained at 37 ± 0.5 °C. To evaluate the sustained release of test drugs, dissolution tests were carried out using Method A of delayed-release dosage forms in USP’s extended-release dosage forms, which involves a pH shift from acidic to buffered conditions.

Acid stage: The test drug was dissolved in 750 mL of 0.1 N hydrochloric acid at 37 ± 0.5 °C for 2 h. After 2 h, withdraw an aliquot of the fluid.

Buffer stage: 250 mL of 0.20 M tribasic sodium phosphate was added to the fluid in the vessel. The final pH was pH was 6.6~6.7.

The samples withdrawn at each time point were filtered using 10 µm porous PVDF sampling filters. An Agilent HPLC 1260 series instrument (Santa Clara, CA, USA), equipped with an ODS silica column (4.6 × 250 mm, 5 µm), was used to determine the concentrations of ruxolitinib, respectively, in the dissolution samples. The HPLC analysis was conducted using a mobile phase with a 4:6 ratio (*v*/*v*) of acetonitrile to purified water (including 0.1% *v*/*v* formic acid), and the flow rate was set to 1.0 mL/min. The column effluent was monitored at 225 nm. Additionally, the dissolution curve of the SR portion of the bilayer tablet was calculated by subtracting the dissolution curve of the Jakavi^®^ at pH 1.2 from the dissolution curve of the entire bilayer tablet, assuming that the dissolution property of the IR portion in the bilayer tablet is similar to that of Jakavi^®^. In this process, the ratio of the IR portion was considered.

$$A_{SR,t}=A_{Total,t}-A_{IR,t}\approx A_{Total}-normalized\;A_{Jak},$$

where *A_SR,t_* and *A_IR,t_* denote the amount released at time *t* from the SR layer and IR layer, respectively; *A_Total,t_* represents the total amount released at same time; and *A_Jak,t_* refers to the amount released from Jakavi^®^ at time *t*.

The following procedure was used to determine the saturated concentration of ruxolitinib in each pH medium. Ruxolitinib phosphate was added to each pH medium at a concentration of 10 mg/mL, which corresponds to 13.2 mg/mL of ruxolitinib phosphate. Each solution was shaken at 37 °C for 24 h and then filtered using 0.2 µm porous PVDF sampling filters. The filtered solution was diluted to a ratio of 1:100. HPLC analysis was conducted to determine the saturated concentration in each pH medium, following the same method used for the dissolution test.

### 2.4. Beagle Bioavailability Study

To confirm the bioavailability and its interchangeability, Jakavi^®^ 20 mg and ruxolitinib bilayer tablets were administered to Beagle. A 20 mg dose of Jakavi^®^ was administered twice a day (0 and 12 h), and a 40 mg dose of the test formulation was administered only once (0 h). The Beagle dogs were purchased from Xi’an Dilepu Biology and Medicine Co., Ltd. (by Saeronbio Inc., Uiwang, Republic of Korea) and kept in a controlled room at a temperature of 23 ± 3 °C, a 12:12 h light/dark cycle, and 55 ± 15% relative humidity. All experiments were conducted according to the protocols approved by the institutional animal care and use committee of KNOTUS Co., Ltd. (Inchon, Republic of Korea).

The experiments were carried out as 2 × 2 crossover studies (*n* = 6 in one group) with a 2-week washout period. A combination of Jakavi^®^ and test drugs (F1, F2, F3, and F4) were tested independently. The reference drug was administered twice daily at 12 h intervals, while the test drug was administered once daily after a 16 h fast. The dogs were allowed free access to water during the pharmacokinetic study, while dog food was provided 8 h following drug administration.

Blood samples (1 mL) were collected from the cephalic vein at designated time points, and the plasma was separated by centrifugation at 3000 rpm for 5 min. All plasma samples were stored at −70 °C until analyzed. After sampling was completed, the frozen plasma samples were transferred to Samyang Holdings’ Instrumental Analysis Team for analysis.

### 2.5. Rat PK Study

A rat PK study was conducted to confirm the restriction of the absorption site of Ruxolitinib. Male Sprague-Dawley rats (7 weeks, weight 240 g, Orient Bio, Seongnam, Korea) were used for in vivo PK studies. Experimental animals were separated into two groups: the gastric dosing group and the intestinal dosing group. Ruxolitinib phosphate was dissolved in saline at a concentration of 5.28 mg/mL. Under anesthesia, the abdomen of SD rats was incised, and 0.3 mL per rat (1.2 mg as Ruxolitinib) of the drug solution was injected into the target organ (gastric, small intestine) using an insulin syringe (*n* = 7/group). After suturing the incision site, the SD rats were recovered for 10 min. Blood samples were collected at appropriate intervals, starting from 30 min and continuing up to 4 h. Blood samples were collected in heparinized tubes and immediately separated into plasma. The separated plasma samples were stored at −70 °C until analysis. All experimental procedures were reviewed and approved by the Instrumental Analysis Team, Samyang Holdings Biopharm group.

### 2.6. Analytical Method

Ruxolitinib in Beagle and rat plasma was analyzed using the LC-MS/MS method. The following procedure was used to determine the plasma concentrations of ruxolitinib. Ruxolitinib phosphate standard was dissolved in methanol to prepare a solution with a concentration of 1000 µg/mL as ruxolitinib. This solution was then serially diluted with methanol to prepare standard solutions for the calibration curve, with final ruxolitinib concentrations of 0.005, 0.01, 0.05, 0.2, 1, 5, 10, and 20 µg/mL. All samples were processed with protein precipitation. In a 2 mL tube, 100 µL of the sample was mixed with 10 µL of internal standard (ruxolitinib-d9, 10 µg/mL) and 400 µL of acetonitrile. The mixture was vortexed and then centrifuged at 4 °C, 12,500 rpm for 7.5 min. The supernatant (200 µL) was transferred to a new LC vial, and 100 µL of mobile phase A (10 mM ammonium formate with 0.1% formic acid in DW) was added. A 5 µL aliquot of this solution was injected into the LC/MS/MS system. The calibration curve was constructed using the peak area ratio of Ruxolitinib to the internal standard.

The LC/MS/MS analysis conditions for Ruxolitinib were as follows.

The 5 μL of the aliquot was injected into the LC-MS/MS system. A 1260 series (Agilent, Santa Clara, CA) with an Agilent 6460 mass spectrometer (Agilent, Santa Clara, CA) and a Synergi™ Polar-RP 2.0 × 50 mm, 4 µm (Phenomenex, Torrance, CA), was used for the study. An isocratic mobile phase consisting of a 45:55 mixture of acetonitrile (ACN) and 10 mM ammonium formate with 0.1% formic acid in distilled water was delivered at a flow rate of 0.25 mL/min. Quantification was achieved using electrospray ionization-MS/MS detection in the positive ion mode for the ruxolitinib and IS. The optimal instrumental parameters were as follows: a capillary voltage of 3.5 kV and a source temperature of 350 °C. The collision energy (CE) was 28 V. Detection of the ions was carried out in the multiple reaction-monitoring mode, and the settings are shown in Table 2.

### 2.7. Pharmacokinetic Analysis and Evaluation of the IVIVC

The calculations of pharmacokinetic parameters and statistical analyses were conducted using the WinNonlin^®^ program (Pharsight Corporation, Mountain View, CA, USA). When the ratios of the area under the plasma concentration–time curve (AUC) and peak concentration (Cmax) of the reference and test drugs fell within the range of 80–125%, the pharmacokinetic profiles of the test drugs were considered to meet the initial development goals.

Following the oral administration of Jakavi^®^, plasma concentration followed a two-compartment pharmacokinetic model. The predicted in vivo fraction of drug absorption was acquired through deconvolution using the Loo–Riegelman method. For IVIVC, the fraction absorbed and the amount fraction released over time were subsequently plotted.

Pharmacokinetic parameters derived from the Loo–Riegelman method, including the central volume of distribution, inter-compartment rate constants, and elimination rate constants, were determined for the IR product Jakavi^®^. This was achieved by fitting a two-compartment model using WinNonlin^®^ (Version 8.5.2) (Pharsight Corporation, Mountain View, CA). The initial parameters for the fitting process were based on intravenous data (*n* = 1) included in the registration filing document of Jakavi^®^. Moreover, to minimize errors in the Loo–Riegelman method, plasma concentrations not obtained at equal intervals six hours post-drug administration were calculated using interpolation. Additionally, the dissolution test results were also corrected to equal intervals of one hour through interpolation.

The relationship between in vitro cumulative dissolution data and in vivo cumulative absorption data was illustrated using linear regression.

For discriminative pharmacokinetic analysis, the plasma concentration of the SR portion of the test drug was determined by subtracting the average blood concentration of the reference drug from the individual blood concentration measured after administering the test drug. The average blood concentration of the reference drug was calculated using data from all reference drug groups (*n* = 16) involved in the experiment, taking into account the immediate-release (IR) ratio of the bilayer tablets in the calculation.

An example of the blood concentration calculation method for the SR portion of formulation F1 (with an IR:SR ratio of 5:35) is illustrated below.

$$Conc_{SR,t}\;of\;F1=Conc_{t}\;of\;F1-Conc_{t_{avg},Jak}\times 5/20$$

In this equation, *Conc_SR,t_* refers the plasma concentration corresponding to the SR layer at time t; *Conc_t_* represents the individual plasma concentration at that same time; and *Conc_t_avg,Jak_* denotes the average plasma concentration of the Jakavi^®^. The value of 5 represents the milligram portion of F1, and 20 indicates the mg dosage of Jakavi^®^.

## 3. Results

### 3.1. The Dissolution Profile of Bilayer Tablets and Reference IR Tablets

**Figure 2:**
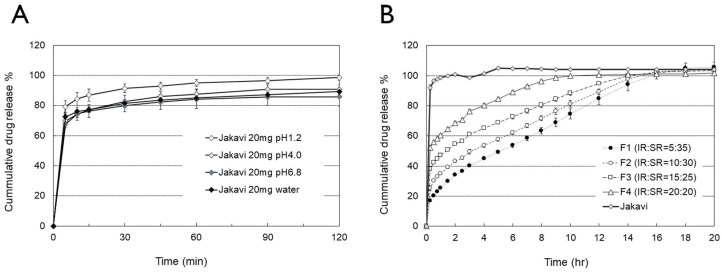
In vitro dissolution profiles of ruxolitinib from different formulations with varying release rates: (**A**) dissolution profile of Jakavi^®^ in pH 1.2, 4.0, and 6.8 buffers, as well as water; (**B**) dissolution profile of bilayer tablets using the buffer transition method A (USP).

The dissolution profiles of reference and bilayer tablets, which consist of varying ratios of IR and SR components, were evaluated. As shown in Figure 2, the in vitro release of ruxolitinib from the reference tablet, Jakavi^®^, was rapid across all tested media. Interestingly, ruxolitinib demonstrated a faster dissolution rate in a pH 1.2 medium compared to other media. This observation suggests that solubility varies depending on the pH or medium used. Specifically, the solubility of ruxolitinib was found to be over 13.4 mg/mL in a pH 1.2 medium, 5.7 mg/mL in a pH 4.0 medium, 1.6 mg/mL in a pH 6.8 medium, and 9.5 mg/mL in water. However approximately 80% of the drug was released within 15 min under all pH conditions, which is an expected result given the solubility of ruxolitinib. In contrast, the dissolution of the bilayer tablet formulation was delayed, depending on the ratio of the SR portion. The release of ruxolitinib using the buffer transition method was found to be 17%, 25%, 38%, and 52% within the initial 15 min for formulations F1, F2, F3, and F4, respectively. In contrast, Jakavi^®^ exhibited a complete release of 92.1% at 15 min. This rapid initial release from the bilayer tablets is expected to contribute to a quick increase in plasma concentration. The dissolution pattern demonstrates the advantages of the bilayer tablet containing both IR and SR layers. As the proportion of the SR layer in the bilayer tablet increased (from F4 to F1), the time for 50% dissolution was delayed to 15 min, 1.5 h, 3 h, and 5 h, respectively.

### 3.2. Discriminative Pharmacokinetic Analysis

In the analytical conditions described above, the optimized separation and detection conditions were achieved for plasma samples. Based on validation results, the lower quantification limit for ruxolitinib in plasma was determined to be 0.4 ng/mL. Figure 2 illustrates the plasma concentration–time curves of ruxolitinib in Beagle dogs following oral administration of Jakavi^®^ and the bilayer tablet formulations F1, F2, F3, and F4. The pharmacokinetic parameters for these formulations are summarized in Table 3. After oral administration of Jakavi^®^, the plasma levels in the Beagle dogs rose quickly, reaching an average maximum concentration (Cmax) of 620 ng/mL at 0.880 h. A wide range of peak plasma concentrations was noted for the bilayer tablets, including the appearance of double-peak phenomena attributed to the SR layer in all bilayer formulations. The most pronounced double peak was observed in F4.

The MRT values of F1, F2, F3, and F4 were 6.55 h, 4.86 h, 4.98 h, and 4.48 h, respectively, which were all significantly increased compared to 1.69 h of IR preparation. This shows that the bilayer layer tablets could effectively maintain the blood concentration of ruxolitinib.

**Figure 3:**
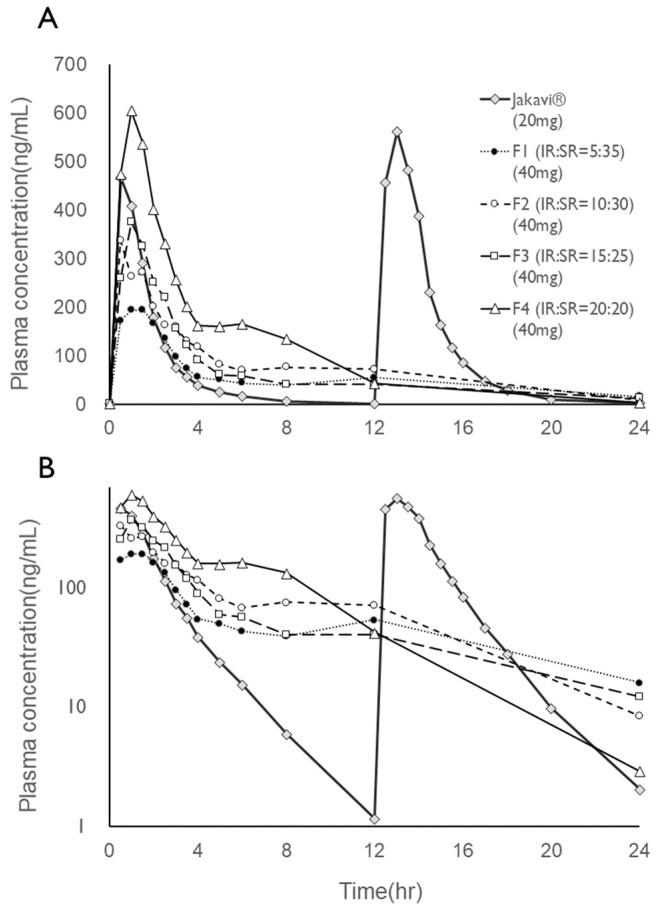
Plasma concentration time profiles ruxolitinib after oral administration of reference and test formulation; (**A**) rectangular scale; (**B**) semi-log scale. The results for the reference formulation, Jakavi^®^, are the combined results of the control group results from four crossover trials for F1, F2, F3, and F4.

Given that the bilayer tablets contained twice the dose of ruxolitinib, the dose-normalized relative bioavailability for F1, F2, F3, and F4 was 23.6%, 35.4%, 41.7%, and 61.8% of that of Jakavi^®^, respectively. Thus, when comparing the dose-normalized relative bioavailability of the bilayer tablets using AUC, it was found to be 58.0%, 80.8%, 68.5%, and 118% for F1, F2, F3, and F4, respectively, compared to Jakavi^®^. The double peak at 5 h, which is considered a result of slow release, was not prominent in F1, F2, and F3, but it was in F4, correlating with the decrease in AUC due to the increased portion of the SR portion. This phenomenon is more clearly illustrated in Figure 3, where the plasma concentration originating from the SR portion is plotted. When comparing plasma concentration normalized to 1 mg of ruxolitinib, F4 exhibited a similar AUC to the immediate-release Jakavi^®^ while delivering a slow release, whereas F1, F2, and F3 indicated an overall decrease in bioavailability.

**Figure 4:**
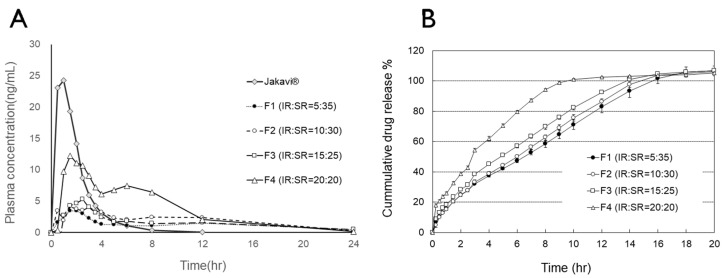
(**A**) Dose-normalized plasma concentration–time profiles of ruxolitinib, which may have originated from the sustained-release (SR) portion of the bilayer tablet. The dose-normalized plasma concentration of the reference drug is also plotted. The results for the reference drug, Jakavi^®^, are the combined results of the control group results from four crossover trials for F1, F2, F3, and F4. (**B**) Release profile of ruxolitinib, potentially from the SR portion of the bilayer tablets.

Interestingly, although the dissolution pattern of the SR portion should be similar, as the same SR formula is used for formulations F1 through F4, as shown in Figure 4, which isolates the dissolution of the SR portion, F4 exhibits a slightly different dissolution pattern compared to F1, F2, and F3. This difference may have an impact on the bioavailability between F4 and the other formulations (F1, F2, and F3).

In terms of bioequivalence, among the four bilayer tablets, only F4 met the criteria for the relative bioavailability point estimates for both AUC and Cmax (Table 3).

### 3.3. IVIVC Evaluation

**Figure 5:**
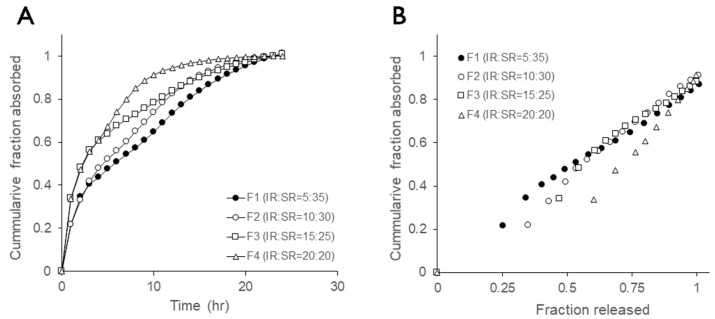
(**A**) Mean cumulative fraction absorbed of ruxolitinib from bilayer tablets calculated by using the Loo–Riegelman approach. (**B**) IVIVC model linear regression plots (y = 0.8883x − 0.0091; correlation coefficient R^2^ = 0.963) of “Fraction released” vs. “Fraction released” for ruxolitinib bilayer tablets.

Since ruxolitinib belongs to BCS Class 1 drugs, it was anticipated that the sustained-release formulation would meet IVIVC Level A. The cumulative fraction absorbed, calculated using the Loo–Riegelman method, and the IVIVC of dissolution are depicted in Figure 5. The cumulative fraction absorbed was observed to be delayed as the sustained-release ratio of the bilayer increased, suggesting that a bilayer strategy with different sustained-release ratios can be effective in developing a sustained-release formulation of ruxolitinib. A Level A IVIVC was investigated using absorbed and released fractions for test formulations, employing Method A for delayed-release dosage forms as per US Pharmacopeia. A linear regression relationship was found between the fraction released and the fraction absorbed for the combined data of the four test drugs (y = 0.8883x − 0.0091; correlation coefficient R^2^ = 0.963), with minor violation for F4.

### 3.4. Rat PK Study

The site-specific absorption or absorption windows may decrease the bioavailability of SR formulation compared to IR formulation. An in vivo rat PK study was conducted to determine whether the site-specific absorption of ruxolitinib exists. Table 4 summarizes the pharmacokinetic parameters of ruxolitinib following administration to the stomach and small intestine in rats. No significant difference was observed between administering the drug in the gastric and small intestinal environments.

## 4. Discussion

In this study, we developed an SR formulation of ruxolitinib aimed to address the pharmacokinetic limitations of its IR counterpart, Jakavi^®^, which necessitates twice-daily dosing due to its short half-life. The results of this study demonstrate the feasibility of a bilayer tablet approach to achieve a sustained drug release profile, potentially enhancing patient compliance and therapeutic outcomes.

The pharmacokinetic profiles of the test formulations demonstrated varied outcomes. Test formulation F1, with a balanced IR:SR ratio, achieved the sustained dosage form of the reference drug, exhibiting comparable AUC values and extended MRT, suggesting that a balanced proportion of IR and SR components can effectively mimic the pharmacokinetic profile of Jakavi^®^ while potentially reducing dosing frequency. The double-peak phenomena observed in some formulations are indicative of complex absorption dynamics, likely influenced by the dissolution characteristics of the SR layer.

Conversely, formulations with higher SR proportions (e.g., test formulation F1, F2, and F3) demonstrated reduced bioavailability, likely due to incomplete absorption within the gastrointestinal tract.

In terms of bioavailability, only test formulation F4 met the criteria for bioequivalence, reflecting the challenges in achieving consistent systemic exposure with higher SR content. These findings underscore the importance of optimizing the SR component to maintain bioavailability and ensure consistent therapeutic outcomes.

The discriminative analysis of the SR portion for plasma concentration and dissolution curve revealed that the SR portion of F1, F2, and F3 have low bioavailability. Considering the high bioavailability of ruxolitinib, this should be defined [16]. Since the difference in solubility in the stomach and small intestine or the presence of specific absorption site and gut wall metabolism is often pointed out as the cause of the decrease in bioavailability due to extended-release [17,18,19], this study examined absorption specificity using rats. However, in rats, similar bioavailability was observed when the drug was administered to the stomach and intestine. However, since absorption specificity by the small intestine site was not examined in this experiment, there may still be an absorption window for the drug at a specific small intestine site, and this may have caused the decrease in bioavailability due to excessive sustained release. It is thought that further research on this aspect will be necessary in the future. The enhanced gut wall metabolism by the slow release of the drug also should be evaluated [19]. To date, decreased bioavailability has been noted for isosorbide dinitrate, propranolol, and diazepam in sustained-release formulations, indicating an increased first-pass effect [20,21,22]. However, these findings have been reported only for specific drugs and cannot be generalized to all sustained-release formulations. Although the mechanism is not yet known and further intensive study is expected, in the case of ruxolitinib, excessive sustained release may lead to a decrease in bioavailability, which should be considered in future development.

As part of an attempt to investigate the validity of the dual-release strategy for the SR dosage form and the cause of the change in bioavailability according to the ratio of the SR layer of the bilayer tablet, discriminative pharmacokinetic analysis and IVIVC were performed on four test formulations. The observation of a Level A correlation between the dissolution rate and absorption rate of the test drug suggests that the decrease in bioavailability observed in F1, F2, and F3 formulations with a high proportion of sustained-release drugs may be due to biological causes other than pharmaceutical factors such as release rate.

An accurate PK/PD model that can be applied to Beagles and humans would be needed to define the optimal plasma concentration profile of the SR formulation of ruxolitinib, but information on such models is currently unknown. Therefore, the best judgment on the optimal formulation would be to rely on an intuitive interpretation of the current Beagle dog results, such as dose-adjusted AUC (T/R ratio %) and Cmax (T/R ratio %). Compared to the reference drug, Jakavi^®^, F4 formulation showed equivalent bioavailability. The dose-adjusted AUC (T/R ratio %) and Cmax (T/R ratio %) were 94.8% and 104.8%, respectively.

It was reported that the IC_50_ of ruxolitinib was 0.065 mg/L, and the target, pSTAT3, was inhibited to a maximum of 60–70% within 1 h, which declined to 25% inhibition at 12–16 h after a single dose of ruxolitinib 25 mg in a human subject. Interestingly, in this study, the period during which ruxolitinib plasma concentrations were greater than IC_50_ was about 4 h of 12 h for Jakavi^®^. Among test formulations, F4 showed the most extended period, i.e., 10.5 h of 24 h. In light of all this information, the F4 formulation should be given the highest priority for consideration as an SR formulation of ruxolitinib.

## 5. Conclusions

In conclusion, the discriminative pharmacokinetic analysis and the IVIVC analysis based on it, which focus on the dissolution and plasma exposure of the IR layer, could effectively evaluate the formulation characteristics of ruxolitinib bilayer tablets composed of various IR and SR weight ratios. Among the test formulations, the F4 formulation was predicted to be the most equivalent to the reference drug Jakavi^®^ in terms of dose-adjusted AUC (T/R ratio %) and Cmax (T/R ratio %). It is also expected that such discriminative pharmacokinetic and IVIVC analysis can be utilized for the further optimization of bilayer formulation after clinical trials.

## Figures and Tables

**Table 1:** Composition of formulations for ruxolitinib bilayer tablet.

		Test Formulations			
	Ingredients (mg)	F1 IR:SR = 5:35	F2 IR:SR = 10:30	F3 IR:SR = 15:25	F4 IR:SR = 20:20
IR layer	Ruxolitinib phosphate	6.6	13.2	19.8	26.4
	Microcrystalline cellulose	44.7	89.5	134.2	178.9
	Lactose monohydrate	46.3	92.5	138.8	185.0
	Sodium starch glycolate	3.1	6.3	9.4	12.5
	Povidone	2.0	4.0	6.0	8.0
	Hydroxypropyl cellulose	2.0	4.0	6.0	8.0
	Colloidal silicon dioxide	1.1	2.1	3.2	4.2
	Magnesium stearate	0.5	1.0	1.5	2.0
	IR layer total	106.3	212.5	318.8	425.0
SR layer	Ruxolitinib phosphate	46.2	39.6	33.0	26.4
	Polyox™ WSR 205	148.8	127.5	106.3	85.0
	Polyox™ WSR N60K	148.8	127.5	106.3	85.0
	polyethylene glycol 6000	87.5	75.0	62.5	50.0
	Hydroxypropyl cellulose	17.2	14.7	12.3	9.8
	Magnesium stearate	6.7	5.7	4.8	3.8
	SR layer total	455.0	390.0	325.0	260.0
	Bi-layer total	561.3	602.5	643.8	685.0
Coating layer	Opadry^®^ 20A	9.8	10.6	11.3	12.0
	Opadry^®^ 200F	16.4	17.6	18.8	20.0

**Table 2:** Summary of monitoring ions and respective parameters for MS detection.

Name	Q1 Mass	Q3 Mass	Fragment (V)	CE (V)
Ruxolitinib	307.1	186.1	158	28
Ruxolitinib-d9 (I.S.)	316.2	186.1	158	28

**Table 3:** Pharmacokinetic parameters of ruxolitinib formulations in Beagle dogs.

	Reference Jakavi^®^	F1 (IR:SR = 5:35)	F2 (IR:SR = 10:30)	F3 (IR:SR = 15:25)	F4 (IR:SR = 20:20)
AUC (h· µg·L^−^^1^)	1139	1323	1840	1560	2690
	(±856)	(±1176)	(±1580)	(±1498)	(±1525)
Cmax (µg·L^−^^1^)	620	293	439	517	766
	(±344)	(±208)	(±273)	(±384)	(±218)
Tmax (h)	0.880	1.5	1	1	1.13
	(±0.452)	(±0.71)	(±0.8)	(±0.56)	(±0.48)
MRT (h)	1.69	6.55	4.86	4.98	4.48
	(±0.69)	(±2.63)	(±2.88)	(±2.62)	(±1.32)
AUC (T/R ratio %)		41.9%	53.0%	75.0%	94.8%
Cmax (T/R ratio %)		24.0%	54.9%	82.2%	104.8%

For the reference drug, pharmacokinetic parameters were calculated using plasma concentrations measured at two intervals: from 0 to 12 h and from 12 to 24 h. The results for the reference drug, Jakavi^®^, are the combined results of the control group results from four crossover trials for F1, F2, F3, and F4 (*n* = 96). The values for Cmax, Tmax, and MRT were calculated as averages of the two time periods, while the AUC was determined by summing the values from both intervals. In the case of the test drug, pharmacokinetic parameters were calculated using plasma concentrations collected over the entire 0 to 24 h period (*n* = 12). AUC (T/R ratio %) and Cmax (T/R ratio %) are the results of crossover trials for the reference drug and F1, F2, F3, and F4 test formulations, respectively.

**Table 4:** Bioavailability parameters of ruxolitinib in rats after gastric and duodenal administration.

Group (N = 7)	Cmax (ng/mL)	AUC (ng/mL·hour)
Gastric administration	74.78 (±30.31)	68.42 (±24.47)
duodenal administration	85.60 (±35.53)	71.43 (±25.57)

## Data Availability

The original contributions presented in this study are included in the article.
